# Selection of *Lactococcus lactis* HY7803 for Glutamic Acid Production Based on Comparative Genomic Analysis

**DOI:** 10.4014/jmb.2011.11022

**Published:** 2020-12-31

**Authors:** Jungmin Lee, Sojeong Heo, Jihoon Choi, Minsoo Kim, Eunji Pyo, Myounghee Lee, Sangick Shin, Jaehwan Lee, Jaehun Sim, Do-Won Jeong

**Affiliations:** 1 Department of Food and Nutrition, Dongduk Women’s University, Seoul 02748, Republic of Korea; 2 R&BD Center, Korea Yakult Co., Ltd., Yongin 17086, Republic of Korea

**Keywords:** Lactic acid bacteria, *Lactococcus lactis*, comparative genome, glutamic acid

## Abstract

Comparative genomic analysis was performed on eight species of lactic acid bacteria (LAB)—*Lactococcus* (*L*.) *lactis, Lactobacillus* (*Lb*.) *plantarum*, *Lb. casei, Lb. brevis, Leuconostoc* (*Leu*.) *mesenteroides, Lb. fermentum, Lb. buchneri*, and *Lb. curvatus*—to assess their glutamic acid production pathways. Glutamic acid is important for umami taste in foods. The only genes for glutamic acid production identified in the eight LAB were for conversion from glutamine in *L. lactis* and *Leu. mesenteroides*, and from glucose via citrate in *L. lactis*. Thus, *L. lactis* was considered to be potentially the best of the species for glutamic acid production. By biochemical analyses, *L. lactis* HY7803 was selected for glutamic acid production from among 17 *L. lactis* strains. Strain HY7803 produced 83.16 pmol/μl glutamic acid from glucose, and exogenous supplementation of citrate increased this to 108.42 pmol/μl. Including glutamic acid, strain HY7803 produced more of 10 free amino acids than *L. lactis* reference strains IL1403 and ATCC 7962 in the presence of exogenous citrate. The differences in the amino acid profiles of the strains were illuminated by principal component analysis. Our results indicate that *L. lactis* HY7803 may be a good starter strain for glutamic acid production.

## Introduction

The flavor of food influences dietary selection and intake. Flavor compounds occur naturally, or are produced during food processing such as cooking or fermentation. Taste is classified into five types based on different taste receptors: sweet, bitter, sour, salty, and umami. Umami is an essential savory taste, contributed by free glutamic acid. The formation of glutamic acid during fermentation, curing, and ripening enhances the umami in foods. Free glutamic acid exists in various foodstuffs and glutamic acid occurs in proteins. The average glutamic acid content of casein in milk, gluten in wheat, glycinin in soybean, and myosin in muscle is 21%–35% [1, 2]. Although glutamic acid in proteins does not contribute umami taste, proteins can be hydrolyzed by proteolytic activity during fermentation and produce high levels of free glutamic acid, which is stable [1].

Lactic acid bacteria (LAB) are involved in fermentation of various foods, such as cheese, sauerkraut, soybean, and wine [3], and are used as starter cultures in dairy, meat, and alcohol fermentation [4]. LAB fermentation through carbohydrate breakdown produces lactic acid as the major metabolite [5]. These cultures also contribute to organoleptic properties, such as texture and flavor, and provide essential micronutrients such as minerals and vitamins. LAB undoubtedly benefit carbohydrate fermentation, enhance nutritional value, enhance production of volatile compounds and bacteriocins, and inhibit the growth of pathogenic and contaminating bacteria. However, not much research has been performed into glutamic acid production by LAB compared with the huge number of functional reports on LAB. Some studies have focused on selection of glutamic acid-producing LAB [6]. Tanous *et al*. proved the existence of the glutamic acid dehydrogenase (*gdh*) gene in LAB, which is responsible for the production of glutamic acid from α-ketoglutarate and ammonia [7]. However, a comprehensive picture of the cellular components and metabolic pathways involved in glutamic acid production has not been obtained for LAB using comparative genomic analysis. Therefore, in this study, we examined the amino acid synthesis, including glutamic acid, of eight LAB species and performed a comparative genomic analysis to compare the metabolic pathways. Additionally, we selected a glutamic acid-producing strain based on biochemical analyses arising from the comparative genomic analysis.

## Materials and Methods

### Comparison of Genomes to Assess Glutamic Acid Production

For comparative genomic analyses, genome sequence data for eight LAB strains was obtained from the NCBI database (http://ncbi.nlm.nih.gov/genomes) (Table 1); the eight species were identified when “glutamic acid” and “LAB” were used as keywords to search the NCBI database. For metabolic pathway analysis based on protein coding genes, genome sequences of the eight strains were uploaded to the Rapid Annotations using Subsystems Technology (RAST) server for SEED-based automated annotation, and subjected to whole-genome sequence-based comparative analysis and Kyoto Encyclopedia of Genes and Genomes pathway analysis [8]. The generated metabolic pathways of the strains were verified using the iPath (ver. 3) module [9]. The Efficient Database framework for comparative Genome Analyses using BLAST score Ratios (EDGAR) was used for core genome, pan-genome and singleton analyses [10]. Further comparative analyses were performed for specific regions and genes-of-interest using the BLASTN, BLASTX, and BLASTP tools.

### Bacterial Strains and Cultures

*Seventeen Lactococcus* (*L*.) *lactis* strains from fermented seafood or dairy products were screened to identify glutamic acid-producing strains (Table 2). *L. lactis* IL1403 (KCTC 3115) [11] and *L. lactis* ATCC 7962 [12] were used as reference strains. *L. lactis* strains were cultured in M17 (Difco, USA) broth or agar supplemented with 0.25% glucose at 30°C for 24 h.

### Screening of Citrate-Using and Glutamic Acid-Producing Strains

Citrate-using strains were detected on Kempler and McKay media [13]. *L. lactis* strains cultured on M17 agar supplemented with 0.25% glucose were transferred to the Kempler and McKay media using toothpicks and incubated at 30°C for 2 days.

### Glutamic Acid Assay

*L. lactis* strains were grown in M17 broth supplemented with 0.25% glucose at 30°C to OD_600_ = 0.6. Then culture supernatant was collected, and 50 μl were used for glutamate assay. Glutamic acid-producing strains were detected using a glutamate assay kit (Sigma-Aldrich, USA) according to the manufacturer’s recommendations. The assay was repeated in triplicate.

### Analysis of Free Amino Acids Including Analysis of Glutamic Acid Production 

*L. lactis* strain HY7803 from an overnight culture was inoculated into M17 broth containing 0.25% glucose (w/v) and 0.5% citrate (w/v) or 0.5% glutamine (w/v) and was cultured to OD_600_ = 1.0. As a negative control, strain HY7803 was inoculated into M17 broth containing 0.25% glucose (w/v). Culture (1 ml) was disrupted by an ultrasonic oscillator (VCX 130, Sonics & Materials, Inc., USA) and then supernatant was obtained by centrifugation (14,000 ×*g*, 1 min, 4°C). Supernatants after sterilization using 0.2-μm syringe filters (Sartorius Stedim Biotech GmbH, Germany) were treated with the AccQ-Fluor Reagent Kit (Waters Corporation, USA) according to the manual. The fluorescent derivatives (10 μl) were used for high-performance liquid chromatography (HPLC). The free amino acid content, including glutamic acid, was calculated using amino acid calibration curves determined at five concentrations with Amino Acid Standard (WAT088122, Waters Corporation). All experiments were conducted three times on two independent samples prepared in the same way.

A JASCO HPLC system (JASCO LC-2000 Plus Series, USA) with a photodiode array detector (detection was at 254 nm in this study), an autosampler (JASCO AS-2055 Plus), and an AccQ-Tag reversed-phase column (4 μm, 150 × 3.9 mm; Waters Corporation) was used for chromatographic separation at 37°C. Eluent A was 10% AccQ-Tag Eluent A Concentrate (Waters Corporation) in water, and eluent B was 60% acetonitrile in water. The gradient elution program began with 100% eluent A at a flow rate of 1 ml/min for the first 9 min, followed by a linear increase to 0%:100% (v/v) eluent A:eluent B for 34 min, and then a decrease to 100%:0% A:B during the next 4 min.

### Statistical Analysis

One-way analysis of variance followed by Duncan’s multiple range test was used to evaluate significant differences between the average values obtained in free amino acid analyses. Values of *p* < 0.05 were considered statistically significant. To visualize the differences between the amino acids produced from sterilized soybeans by the inoculated bacteria, principal component analysis (PCA) was applied with maximum variation rotation. All statistical analysis was performed using the SPSS software package (version 22.0; SPSS, IBM, USA).

## Results and Discussion

### Genomic Analysis of Glutamic Acid Production by LAB

*L. lactis*, *Lactobacillus* (*Lb*.) *plantarum*, *Lb. casei*, *Lb. brevis*, *Lb. fermentum*, *Lb. buchneri*, *Lb. curvatus*, and *Leuconostoc* (*Leu*.) *mesenteroides* were associated with glutamic acid production in the NCBI search engine. Therefore, published, complete genomes of these species were used for comparative genomic analysis (Table 1).

**Fig. 1:**
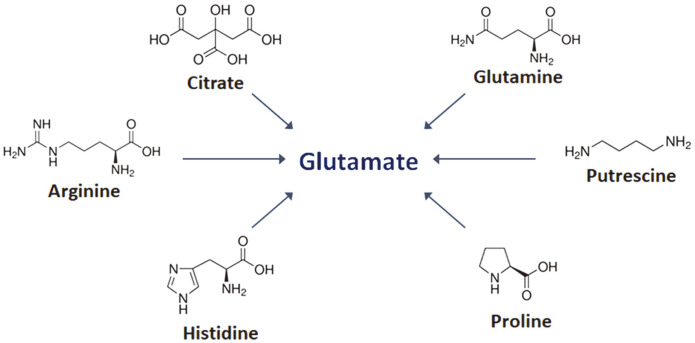
Precursors for glutamate biosynthesis.

**Fig. 2:**
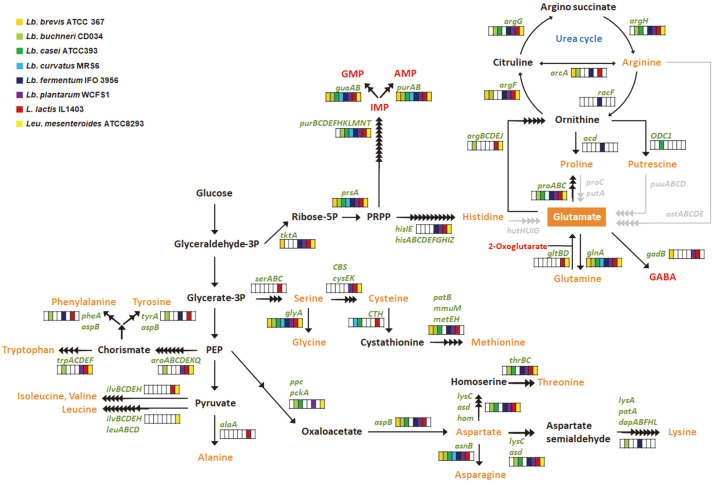
Predicted amino acid synthesis pathways in lactic acid bacteria. The names of enzyme-encoding genes are depicted in green. Metabolites involved in fermentation pathways are depicted in orange. Black arrows correspond to potential enzymatic reactions catalyzed by gene products encoded in eight lactic acid bacterial genomes. Putative inactive pathways are depicted with gray arrows.

**Fig. 3:**
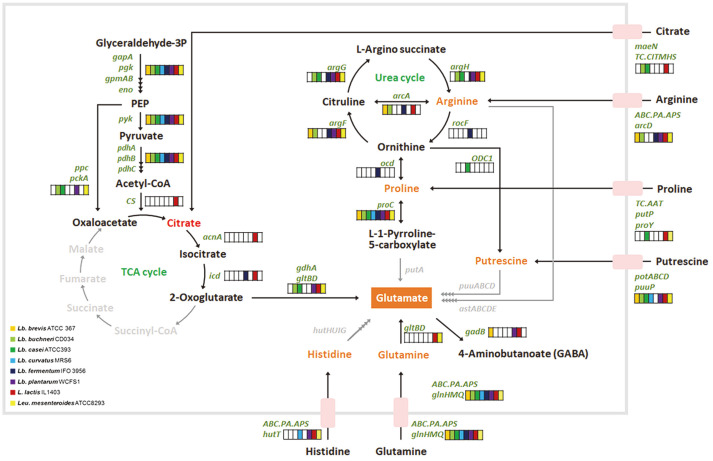
Predicted membrane transport systems related to glutamate synthesis pathways in lactic acid bacteria. The names of enzyme-encoding genes are depicted in green. Metabolites involved in fermentation pathways are depicted in orange. Black arrows correspond to potential enzymatic reactions catalyzed by gene products encoded in eight lactic acid bacterial genomes. Putative inactive pathways are depicted with gray arrows.

There are five pathways for glutamic acid production—from the precursors citrate, glutamine, histidine, proline, and putrescine (Fig. 1). Glutamic acid can be produced from arginine via proline or putrescine (Figs. 1 and 2). Other than citrate, these precursors are amino acids or an amino acid derivative. So, in silico prediction of the amino acid biosynthesis pathways in the eight LAB species was performed (Fig. 2 and Table S1). The genome of *L. lactis* IL1403 contained the genes for synthesis of 11 amino acids; *Lb. buchneri* CD034 contained genes for the synthesis of seven amino acids. None of the genomes of the eight species possessed genes for the synthesis of glutamic acid from histidine or putrescine. Proline, a potential precursor of glutamic acid, was not synthesized directly by any of the species, but it could be synthesized from arginine. None of the genomes included genes for arginine synthesis, but they included the gene encoding a transporter for intake of proline and arginine. None of the genomes contained genes for the synthesis of glutamine, but the genomes of *L. lactis* IL1403 and *Leu. mesenteroides* ATCC 8293 possessed genes for synthesis of glutamic acid from glutamine. Glutamine may be obtained from exogenous sources; transporters of glutamine were identified in all eight genomes (Fig. 3). Citrate could be synthesized from pyruvate in *L. lactis* strain IL1403, and this bacterium has genes encoding a specific transporter for exogenous citrate (Fig. 3).

In summary, the genomes of *L. lactis* IL1403 and *Leu. mesenteroides* ATCC 8293 possessed genes required for the synthesis of glutamic acid from glutamine, and the genome of *L. lactis* IL1403 possessed genes required for the synthesis of glutamic acid from citrate. Therefore, based on comparative genomic analysis, we suggest that *L. lactis* IL1403 may produce glutamic acid.

### Screening of Glutamic Acid-Producing Strains

We screened 17 *L. lactis* strains, which were originally isolated from fermented seafood and dairy products, to select glutamic acid-producing strains based on citrate-utilization activity and a glutamic acid assay kit (Table 2). Three strains—KS3075, HY7803, and Y25—used citrate, as did reference strain ATCC 7962. Among them, strain HY7803 produced the most glutamic acid.

### Effect of Citrate and Glutamine as External Materials on Glutamic Acid Production 

Based on comparative genomic analysis (see section 3.1), citrate and glutamine might be used as precursors for glutamic acid production by *L. lactis*. We quantitatively determined glutamic acid production by *L. lactis* HY7803 using HPLC; 0.5% (w/v) citrate or 0.5% (w/v) glutamine were added to the culture medium to test their effect on glutamic acid production. For strain HY7803, the presence of citrate increased the glutamic acid production to 108.42 ± 0.47 pmol/μl from 83.16 ± 2.19 pmol/μl in the control (no added citrate). For strain IL1403, citrate decreased the glutamic acid production to 59.08 ± 3.26 pmol/μl from 75.15 ± 3.89 pmol/μl in the control (Table 3). Exogenous glutamine decreased the glutamic acid production in three strains. Through these experiments, we showed that *L. lactis* HY7803 may be able to enhance glutamic acid production during fermentation, especially in the presence of citrate as an exogenous precursor.

### Free Amino Acid Production by Three *L. lactis* Strains

Amino acids, including glutamic acid, affect the sensory properties of food, so we checked the free amino acid production by *L. lactis* strains. Sixteen free amino acids were identified in the M17 broth (Table 3). Citrate significantly changed the free amino acid production by strains HY7803, ATCC 7962, and IL1403, while glutamine generally decreased the amino acid production. These results indicated that glutamine was not an efficient exogenous material for amino acid production. Under citrate exposure, 10 amino acids were produced by strain HY7803 at higher levels than the other two strains (Table 3).

**Fig. 4:**
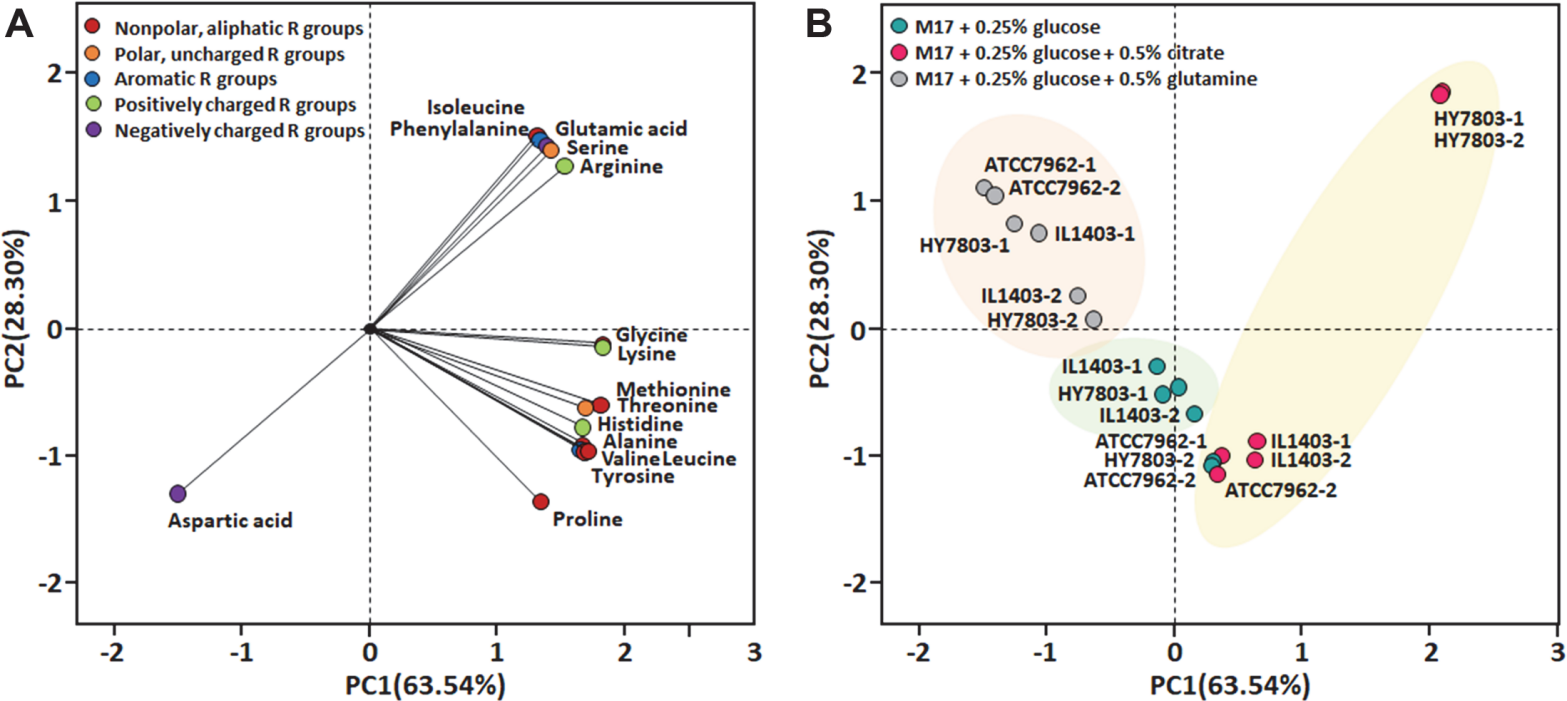
Principal component analysis loadings for three Lactococcus lactis strains grown in the presence of citrate, glutamine, or neither for (A) amino acids identified (represented according to their classifications), and (B) factor scores (numbers indicate independent samples).

Statistics on the 16 free amino acids produced by the three *L. lactis* strains were subjected to PCA (Fig. 4). In a PCA factor loading plot, isoleucine, phenylalanine, serine, arginine, and glutamic acid were located in the positive part of the PC2 dimension. The 15 free amino acids other than aspartic acid were positively correlated with PC1 (Fig. 4A). The PCA scores for the three *L. lactis* strains cultured in the presence of citrate or glutamine are shown in Fig. 4B. The factor scores for cultures grown in the presence of glutamine clustered separately from those of control samples. The data indicate that exogenous glutamine negatively affects amino acid production in *L. lactis*. In the presence of citrate, strains ATCC 7962 and IL1403 showed similar scores. Strain HY7803 exhibited the highest production of arginine, isoleucine, phenylalanine, serine, and glutamic acid by significant amounts, and these results affect the factor scores. Therefore, strain HY7803 might be used to produce amino acids, including glutamic acid.

In conclusions, most approaches to selecting starter candidates screen large libraries of bacteria [14-18]. However, the current study aimed to select suitable bacteria based on comparative genomic analysis. Such analysis of eight LAB species suggested that *L. lactis* may be suitable for glutamic acid production. Glutamic acid production through fermentation was showed low yield and purity compared with traditional methods such as extraction. However, fermented products added *L. lactis* for glutamic acid enhancement would have an additional advantage such as health benefits, as well as safety aspects. Biochemical analysis identified *L. lactis* strain HY7803 as a starter candidate; *L. lactis* HY7803 exhibited glutamic acid production, and citrate induced the glutamic acid production. Therefore, we suggest that *L. lactis* HY7803 displays desirable properties of a starter culture for enhancing taste properties in fermented foods.

## Supplemental Materials



Supplementary data for this paper are available on-line only at http://jmb.or.kr.

## Figures and Tables

**Table 1:** Lactic acid bacteria used for genomic analysis in this study.

Species	Strain	NCBI Accession No.
*Lactobacillus brevis*	ATCC 367	NC_008497.1
*Lactobacillus buchneri*	CD034	NC_018610
*Lactobacillus casei*	ATCC 393	NZ_AP012544
*Lactobacillus curvatus*	MRS6	NZ_CP022474
*Lactobacillus fermentum*	IFO 3956	NC_010610
*Lactobacillus plantarum*	WCFS1	NC_004567
*Lactococcus lactis*	IL1403	NC_002662
*Leuconostoc mesenteroides*	ATCC 8293	NZ_008531.1

**Table 2:** Citrate use and glutamate production by *Lactococcus lactis* isolates.

Strain	Citrate fermentation	Glutamate concentration^a^ (ng/μl)	Reference	Origin
ATCC 7962	+	9.74	[12]	Control
IL1403	+	16.91	[11]	Control
KS3075	+	5.33	[19]	Fermented seafood
HY7803	+	17.31	This study	Dairy product
Y2	−	8.66	This study	Dairy product
Y4	−	7.04	This study	Dairy product
Y6	−	6.91	This study	Dairy product
Y7	−	7.16	This study	Dairy product
Y8	−	9.35	This study	Dairy product
Y19	−	9.38	This study	Dairy product
Y20	−	7.92	This study	Dairy product
Y21	−	6.81	This study	Dairy product
Y22	−	7.12	This study	Dairy product
Y23	−	6.51	This study	Dairy product
Y25	+	9.19	This study	Dairy product
Y26	+	10.40	This study	Dairy product
Y28	−	7.56	This study	Dairy product
Y32	−	7.37	This study	Dairy product
Y33	−	6.98	This study	Dairy product

^a^Determined using a glutamate assay kit (Sigma-Aldrich).

**Table 3:** Free amino acid profiles of three *L. lactis* strains under citrate or glutamine pressure. (unit: pmol/μl)

	*L. lactis* HY7803			*L. lactis* ATCC7962			*L. lactis* IL1403		
		
	Control	Citrate	Glutamine	Control	Citrate	Glutamine	Control	Citrate	Glutamine
Aspartic acid	26.70^bc^	49.27^f^	19.63^a^	30.52^cd^	31.52^cd^	23.25^ab^	39.03^e^	73.55^g^	32.81^d^
Glutamic acid	83.16^de^	108.42^f^	60.65^a^	80.61^cde^	88.01^e^	62.54^ab^	75.15^cd^	59.08^a^	71.67^bc^
Serine	80.54^e^	99.08^f^	66.12^bcd^	58.97^b^	69.82^cd^	47.35^a^	73.65^de^	82.47^e^	61.24^bc^
Histidine	30.65^b^	35.89^cd^	29.49^b^	31.82^bc^	35.28^cd^	21.66^a^	30.71^b^	39.27^d^	25.22^a^
Glycine	55.05^bc^	68.52^d^	43.19^a^	52.07^b^	60.65^c^	42.31^a^	60.80^c^	68.55^d^	50.83^b^
Threonine	80.28^de^	88.48^e^	64.78^b^	67.32^bc^	76.60^cd^	51.62^a^	71.64^bcd^	74.15^bcd^	52.82^a^
Arginine	16.02^d^	20.20^e^	13.07^b^	11.95^b^	13.26^bd^	9.29^a^	15.67^cd^	13.94^bcd^	8.83^a^
Alanine	80.77^b^	96.19^c^	63.21^a^	99.59^c^	86.36^b^	66.39^a^	82.09^b^	98.97^c^	68.28^a^
Tyrosine	51.33^b^	61.97^c^	40.77^a^	52.06^b^	60.00^c^	37.67^a^	52.55^b^	50.78^b^	40.24^a^
Valine	69.27^cd^	76.06^d^	54.76^a^	68.09^cd^	71.90^cd^	50.41^a^	65.02^bc^	70.96^cd^	55.84^ab^
Methionine	20.03^abc^	25.60^d^	16.72^a^	26.56^d^	22.68^cd^	15.96^a^	21.19^bc^	23.99^cd^	17.37^ab^
Phenylalanine	76.14^b^	111.51^d^	60.66^a^	77.17^bc^	88.58^c^	56.53^a^	79.13^bc^	77.68^bc^	60.83^a^
Isoleucine	53.20^d^	63.76^e^	41.61^abc^	48.83^cd^	53.74^d^	36.06^a^	46.60^bcd^	46.71^bcd^	39.83^ab^
Leucine	115.86^cd^	143.87^e^	91.19^ab^	133.64^de^	116.84^cd^	82.73^a^	107.42^bc^	111.41^c^	91.17^ab^
Lysine	42.99^de^	46.54^ef^	33.77^ab^	43.35^de^	36.32^bc^	31.29^a^	39.24^cd^	50.47^f^	36.01^bc^
Proline	38.84^bc^	35.50^bc^	31.02^ab^	39.74^c^	39.05^bc^	26.40^a^	34.74^bc^	41.11^c^	33.35^abc^

*Lactococcus lactis* inoculated into M17 broth supplemented with 0.25% glucose (control). Citrate (0.5% w/v) or glutamine (0.5% w/v) was used as external pressure. Different superscripts within a row denote a significant difference between mean values (*p* < 0.05) according to Duncan’s multiple range test.
