# Supplementary material for: Selection of Lactococcus lactis HY7803 for Glutamic Acid Production Based on Comparative Genomic Analysis
Source: J Microbiol Biotechnol. 2020 Dec 31;31(2):298–303. doi: 10.4014/jmb.2011.11022 (PMC9705870; doi:10.4014/jmb.2011.11022)
Supplement: Supplementary file 1 [file jmb-31-2-298-supple.pdf]

Table S1. Predicted amino acids synthetic pathways in lactic acid bacteria.

Gene	Other name(s)	*Lb. brevis* ATCC 367	*Lb. buchneri* CD034	*Lb. casei* ATCC 393	*Lb. curvatus* MRS6	*Lb. fermentum* IFO 3956	*Lb. plantarum* WCFS1	*L. lactis* IL1403	*Leu. mesenteroides* ATCC 8293	KEGG ID	Enzyme full name
**Histidine**											
tktA|tktB		LVIS_RS18460				LAF_RS09780	lp_0489	L0043	LEUM_RS05705	K00615	Transketolase
tktA|tktB							lp_1083			K00615	Transketolase
tktA|tktB							lp_3135			K00615	Transketolase
tktA|tktB							lp_3538			K00615	Transketolase
PRPS|prsA		LVIS_RS13875	LBUCD034_RS08065	LBCZ_RS12200	CG419_RS01975	LAF_RS01120	lp_0471	L25614	LEUM_RS03400	K00948	Ribose-phosphate pyrophosphokinase
PRPS|prsA							lp_2166			K00948	Ribose-phosphate pyrophosphokinase
hisG			LBUCD034_RS03655			LAF_RS04455	lp_2560	L0066	LEUM_RS10520	K00765	ATP phosphoribosyltransferase
hisZ			LBUCD034_RS03650			LAF_RS04450		L0341	LEUM_RS10520	K02502	ATP phosphoribosyltransferase regulatory subunit
hisZ							lp_2561			K02502	ATP phosphoribosyltransferase regulatory subunit
hisE			LBUCD034_RS03685	LBCZ_RS06665		LAF_RS04490	lp_2552		LEUM_RS07575	K01523	Phosphoribosyl-ATP pyrophosphohydrolase
hisIE				LBCZ_RS06670				L0072		K11755	Phosphoribosyl-ATP pyrophosphohydrolase / phosphoribosyl-AMP cyclohydrolase
hisI			LBUCD034_RS03680			LAF_RS04485	lp_2553		LEUM_RS07580	K01496	Phosphoribosyl-AMP cyclohydrolase
hisA			LBUCD034_RS03670	LBCZ_RS06680		LAF_RS04475	lp_2556	L0070	LEUM_RS07590	K01814	Phosphoribosylformimino-5-aminoimidazole carboxamide ribotide isomerase
hisH			LBUCD034_RS03665	LBCZ_RS06685		LAF_RS04435	lp_2557	L0069	LEUM_RS07595	K02501	Imidazole glycerol-phosphate synthase subunit HisH
hisH						LAF_RS04470				K02501	Imidazole glycerol-phosphate synthase subunit HisH
hisF			LBUCD034_RS03675	LBCZ_RS06675		LAF_RS04480	lp_2554	L0071	LEUM_RS07585	K02500	Imidazole glycerol-phosphate synthase subunit HisF
hisF						LAF_RS04440				K02500	Imidazole glycerol-phosphate synthase subunit HisF
hisB			LBUCD034_RS03660	LBCZ_RS06690		LAF_RS04465	lp_2558	L0068	LEUM_RS07600	K01693	Imidazoleglycerol-phosphate dehydratase
hisC			LBUCD034_RS03690	LBCZ_RS06710	CG419_RS06545	LAF_RS04495	lp_2551	L0065	LEUM_RS07570	K00817	Histidinol-phosphate aminotransferase
hisC			LBUCD034_RS09100							K00817	Histidinol-phosphate aminotransferase
-	histidinol-phosphatase			LBCZ_RS05625		LAF_RS04445	lp_2563	L37351	LEUM_RS07625	K04486	Histidinol-phosphatase (PHP family)
hisD			LBUCD034_RS03225			LAF_RS04460	lp_2559	L0067	LEUM_RS07605	K00013	Histidinol dehydrogenase
**Serine**											
serA|PHGDH		LVIS_RS12240	LBUCD034_RS00295	LBCZ_RS06870	CG419_RS05575	LAF_RS00915	lp_0203	L0084	LEUM_RS02170	K00058	D-3-phosphoglycerate dehydrogenase / 2-oxoglutarate reductase
serA|PHGDH		LVIS_RS19555	LBUCD034_RS00330			LAF_RS04525	lp_2790	L51032	LEUM_RS02455	K00058	D-3-phosphoglycerate dehydrogenase / 2-oxoglutarate reductase
serA|PHGDH			LBUCD034_RS01495			LAF_RS05185			LEUM_RS06020	K00058	D-3-phosphoglycerate dehydrogenase / 2-oxoglutarate reductase
serA|PHGDH			LBUCD034_RS04220			LAF_RS10280			LEUM_RS06435	K00058	D-3-phosphoglycerate dehydrogenase / 2-oxoglutarate reductase
serA|PHGDH			LBUCD034_RS08150						LEUM_RS07150	K00058	D-3-phosphoglycerate dehydrogenase / 2-oxoglutarate reductase
serA|PHGDH			LBUCD034_RS10685							K00058	D-3-phosphoglycerate dehydrogenase / 2-oxoglutarate reductase
serC|PSAT1			LBUCD034_RS01500			LAF_RS04520	lp_0204	L0083	LEUM_RS07155	K00831	Phosphoserine aminotransferase
serB|PSPH								L0085		K01079	Phosphoserine phosphatase
**Glycine**											
serA|PHGDH		LVIS_RS12240	LBUCD034_RS00295	LBCZ_RS06870	CG419_RS05575	LAF_RS00915	lp_0203	L0084	LEUM_RS02170	K00058	D-3-phosphoglycerate dehydrogenase / 2-oxoglutarate reductase
serA|PHGDH		LVIS_RS19555	LBUCD034_RS00330			LAF_RS04525	lp_2790	L51032	LEUM_RS02455	K00058	D-3-phosphoglycerate dehydrogenase / 2-oxoglutarate reductase
serA|PHGDH			LBUCD034_RS01495			LAF_RS05185			LEUM_RS06020	K00058	D-3-phosphoglycerate dehydrogenase / 2-oxoglutarate reductase
serA|PHGDH			LBUCD034_RS04220			LAF_RS10280			LEUM_RS06435	K00058	D-3-phosphoglycerate dehydrogenase / 2-oxoglutarate reductase
serA|PHGDH			LBUCD034_RS08150						LEUM_RS07150	K00058	D-3-phosphoglycerate dehydrogenase / 2-oxoglutarate reductase
serA|PHGDH			LBUCD034_RS10685							K00058	D-3-phosphoglycerate dehydrogenase / 2-oxoglutarate reductase
serC|PSAT1			LBUCD034_RS01500			LAF_RS04520	lp_0204	L0083	LEUM_RS07155	K00831	Phosphoserine aminotransferase
serB|PSPH								L0085		K01079	Phosphoserine phosphatase
glyA|SHMT		LVIS_RS17810	LBUCD034_RS01500	LBCZ_RS05405	CG419_RS04760	LAF_RS02470	lp_2375	L0082	LEUM_RS03840	K00600	Glycine hydroxymethyltransferase
**Threonine**											
ppc			LBUCD034_RS04290						LEUM_RS08250	K01595	Phosphoenolpyruvate carboxylase
pckA				LBCZ_RS08740			lp_3418			K01610	Phosphoenolpyruvate carboxykinase (ATP)
aspB		LVIS_RS15700	LBUCD034_RS05885	LBCZ_RS06920		LAF_RS06085	lp_1739	L0098		K00812	Aspartate aminotransferase
aspB										K00812	Aspartate aminotransferase
lysC			LBUCD034_RS10075	LBCZ_RS00195		LAF_RS04995	lp_0979	L0097	LEUM_RS03820	K00928	Aspartate kinase
lysC			LBUCD034_RS11660	LBCZ_RS09995			lp_2308			K00928	Aspartate kinase
asd			LBUCD034_RS10040	LBCZ_RS00205		LAF_RS05030	lp_1346	L66199	LEUM_RS09445	K00133	Aspartate-semialdehyde dehydrogenase
hom			LBUCD034_RS11665	LBCZ_RS09990		LAF_RS04845	lp_0571	L0090	LEUM_RS03830	K00003	Homoserine dehydrogenase
thrB1		LVIS_RS14215	LBUCD034_RS07540	LBCZ_RS09980		LAF_RS06570	lp_0572	L0091	LEUM_RS03825	K00872	Homoserine kinase
thrC			LBUCD034_RS11670	LBCZ_RS09985		LAF_RS06560	lp_2758	L0092	LEUM_RS03835	K01733	Threonine synthase
**Cysteine**											
serA|PHGDH		LVIS_RS12240	LBUCD034_RS00295	LBCZ_RS06870	CG419_RS05575	LAF_RS00915	lp_0203	L0084	LEUM_RS02170	K00058	D-3-phosphoglycerate dehydrogenase / 2-oxoglutarate reductase
serA|PHGDH		LVIS_RS19555	LBUCD034_RS00330			LAF_RS04525	lp_2790	L51032	LEUM_RS02455	K00058	D-3-phosphoglycerate dehydrogenase / 2-oxoglutarate

Gene	Other name(s)	*Lb. brevis* ATCC 367	*Lb. buchneri* CD034	*Lb. casei* ATCC 393	*Lb. curvatus* MRS6	*Lb. fermentum* IFO 3956	*Lb. plantarum* WCFS1	*L. lactis* IL1403	*Leu. mesenteroides* ATCC 8293	KEGG ID	Enzyme full name
											reductase
serA|PHGDH			LBUCD034_RS01495			LAF_RS05185			LEUM_RS06020	K00058	D-3-phosphoglycerate dehydrogenase / 2-oxoglutarate reductase
serA|PHGDH			LBUCD034_RS04220			LAF_RS10280			LEUM_RS06435	K00058	D-3-phosphoglycerate dehydrogenase / 2-oxoglutarate reductase
serA|PHGDH			LBUCD034_RS08150						LEUM_RS07150	K00058	D-3-phosphoglycerate dehydrogenase / 2-oxoglutarate reductase
serA|PHGDH			LBUCD034_RS10685							K00058	D-3-phosphoglycerate dehydrogenase / 2-oxoglutarate reductase
serC|PSAT1			LBUCD034_RS01500			LAF_RS04520	lp_0204	L0083	LEUM_RS07155	K00831	Phosphoserine aminotransferase
serB|PSPH								L0085		K01079	Phosphoserine phosphatase
cysE							lp_0254	L0087	LEUM_RS04095	K00640	Serine O-acetyltransferase
cysE									LEUM_RS06295	K00640	Serine O-acetyltransferase
cysE									LEUM_RS09935	K00640	Serine O-acetyltransferase
cysK				LBCZ_RS01680		LAF_RS02010	lp_0256	L0088	LEUM_RS06285	K01738	Cysteine synthase
cysK						LAF_RS05640		L0089		K01738	Cysteine synthase
CBS										K01697	Cystathionine beta-synthase
**Methionine**											
serA|PHGDH		LVIS_RS12240	LBUCD034_RS00295	LBCZ_RS06870	CG419_RS05575	LAF_RS00915	lp_0203	L0084	LEUM_RS02170	K00058	D-3-phosphoglycerate dehydrogenase / 2-oxoglutarate reductase
serA|PHGDH		LVIS_RS19555	LBUCD034_RS00330			LAF_RS04525	lp_2790	L51032	LEUM_RS02455	K00058	D-3-phosphoglycerate dehydrogenase / 2-oxoglutarate reductase
serA|PHGDH			LBUCD034_RS01495			LAF_RS05185			LEUM_RS06020	K00058	D-3-phosphoglycerate dehydrogenase / 2-oxoglutarate reductase
serA|PHGDH			LBUCD034_RS04220			LAF_RS10280			LEUM_RS06435	K00058	D-3-phosphoglycerate dehydrogenase / 2-oxoglutarate reductase
serA|PHGDH			LBUCD034_RS08150						LEUM_RS07150	K00058	D-3-phosphoglycerate dehydrogenase / 2-oxoglutarate reductase
serA|PHGDH			LBUCD034_RS10685							K00058	D-3-phosphoglycerate dehydrogenase / 2-oxoglutarate reductase
serC|PSAT1			LBUCD034_RS01500			LAF_RS04520	lp_0204	L0083	LEUM_RS07155	K00831	Phosphoserine aminotransferase
serB|PSPH								L0085		K01079	Phosphoserine phosphatase
cysE							lp_0254	L0087	LEUM_RS04095	K00640	Serine O-acetyltransferase
cysE									LEUM_RS06295	K00640	Serine O-acetyltransferase
cysE									LEUM_RS09935	K00640	Serine O-acetyltransferase
cysK				LBCZ_RS01680		LAF_RS02010	lp_0256	L0088	LEUM_RS06285	K01738	Cysteine synthase
cysK						LAF_RS05640		L0089		K01738	Cysteine synthase
CBS										K01697	Cystathionine beta-synthase
CTH				LBCZ_RS07860	CG419_RS03550			L0102		K01758	Cystathionine gamma-lyase
patB|malY		LVIS_RS13000	LBUCD034_RS03160	LBCZ_RS04085	CG419_RS02850	LAF_RS05465	lp_2751	L177593		K14155	Cysteine-S-conjugate beta-lyase
patB|malY				LBCZ_RS08505			lp_2888			K14155	Cysteine-S-conjugate beta-lyase
patB|malY							lp_3517			K14155	Cysteine-S-conjugate beta-lyase
mmuM						LAF_RS04860	lp_1298			K00547	Homocysteine S-methyltransferase
metH|MTR							lp_1374			K00548	5-methyltetrahydrofolate--homocysteine methyltransferase
metE		LVIS_RS12800	LBUCD034_RS01745	LBCZ_RS02080		LAF_RS01780	lp_1375	L0100	LEUM_RS08745	K00549	5-methyltetrahydropteroyltriglutamate--homocysteine methyltransferase
metE			LBUCD034_RS11390	LBCZ_RS02790		LAF_RS09130	lp_1856	L124252	LEUM_RS08770	K00549	5-methyltetrahydropteroyltriglutamate--homocysteine methyltransferase
metE							lp_3283		LEUM_RS08780	K00549	5-methyltetrahydropteroyltriglutamate--homocysteine methyltransferase
**Alanine**											
alaA								L162604		K14260	Alanine-synthesizing transaminase
alaA								L38687		K14260	Alanine-synthesizing transaminase
**Aspartate**											
ppc			LBUCD034_RS04290						LEUM_RS08250	K01595	Phosphoenolpyruvate carboxylase
pckA				LBCZ_RS08740			lp_3418			K01610	Phosphoenolpyruvate carboxykinase (ATP)
aspB		LVIS_RS15700	LBUCD034_RS05885	LBCZ_RS06920		LAF_RS06085	lp_1739	L0098		K00812	Aspartate aminotransferase
aspB										K00812	Aspartate aminotransferase
**Asparagine**											
ppc			LBUCD034_RS04290						LEUM_RS08250	K01595	Phosphoenolpyruvate carboxylase
pckA				LBCZ_RS08740			lp_3418			K01610	Phosphoenolpyruvate carboxykinase (ATP)
aspB		LVIS_RS15700	LBUCD034_RS05885	LBCZ_RS06920		LAF_RS06085	lp_1739	L0098		K00812	Aspartate aminotransferase
aspB										K00812	Aspartate aminotransferase
asnB|ASNS		LVIS_RS14280	LBUCD034_RS06670	LBCZ_RS10055	CG419_RS07250	LAF_RS03755	lp_0980	L00396		K01953	Asparagine synthase (glutamine-hydrolysing)
asnB|ASNS		LVIS_RS15030				LAF_RS10455	lp_3085	L0095		K01953	Asparagine synthase (glutamine-hydrolysing)
asnB|ASNS		LVIS_RS18860								K01953	Asparagine synthase (glutamine-hydrolysing)
**Lysine**											
ppc			LBUCD034_RS04290						LEUM_RS08250	K01595	Phosphoenolpyruvate carboxylase
pckA				LBCZ_RS08740			lp_3418			K01610	Phosphoenolpyruvate carboxykinase (ATP)
aspB		LVIS_RS15700	LBUCD034_RS05885	LBCZ_RS06920		LAF_RS06085	lp_1739	L0098		K00812	Aspartate aminotransferase
aspB										K00812	Aspartate aminotransferase
lysC			LBUCD034_RS10075	LBCZ_RS00195		LAF_RS04995	lp_0979	L0097	LEUM_RS03820	K00928	Aspartate kinase
lysC			LBUCD034_RS11660	LBCZ_RS09995			lp_2308			K00928	Aspartate kinase
asd			LBUCD034_RS10040	LBCZ_RS00205		LAF_RS05030	lp_1346	L66199	LEUM_RS09445	K00133	Aspartate-semialdehyde dehydrogenase
dapA			LBUCD034_RS10040	LBCZ_RS00175		LAF_RS05015	lp_2685	L0093	LEUM_RS03265	K01714	4-hydroxy-tetrahydrodipicolinate synthase

Gene	Other name(s)	*Lb. brevis* ATCC 367	*Lb. buchneri* CD034	*Lb. casei* ATCC 393	*Lb. curvatus* MRS6	*Lb. fermentum* IFO 3956	*Lb. plantarum* WCFS1	*L. lactis* IL1403	*Leu. mesenteroides* ATCC 8293	KEGG ID	Enzyme full name
dapB			LBUCD034_RS10050	LBCZ_RS00170		LAF_RS05020	lp_1874	L0094	LEUM_RS03265	K00215	4-hydroxy-tetrahydrodipicolinate reductase
dapB										K00215	4-hydroxy-tetrahydrodipicolinate reductase
dapH|dapD			LBUCD034_RS10065			LAF_RS05005				K05822	Tetrahydrodipicolinate N-acetyltransferase
patA			LBUCD034_RS00995	LBCZ_RS03615		LAF_RS00160	lp_2684	L56488	LEUM_RS06015	K00841	Aminotransferase
patA			LBUCD034_RS01545			LAF_RS00730			LEUM_RS06600	K00841	Aminotransferase
patA			LBUCD034_RS10045			LAF_RS05025			LEUM_RS06720	K00841	Aminotransferase
patA									LEUM_RS08960	K00841	Aminotransferase
dapL			LBUCD034_RS10060			LAF_RS05010	lp_2263	L80177	LEUM_RS03260	K05823	N-acetyldiaminopimelate deacetylase
dapF			LBUCD034_RS10080	LBCZ_RS00200		LAF_RS04990	lp_2185			K01778	Diaminopimelate epimerase
lysA			LBUCD034_RS10070	LBCZ_RS00190		LAF_RS05000	lp_1713	L0121	LEUM_RS03250	K01586	Diaminopimelate decarboxylase
**Tryptophan**											
aroF|aroG|aroH								L0063		K01626	3-deoxy-7-phosphoheptulonate synthase
aroF|aroG|aroH								L0064		K01626	3-deoxy-7-phosphoheptulonate synthase
AROA2|aroA			LBUCD034_RS11855		CG419_RS05415		lp_1085		LEUM_RS05695	K03856	3-deoxy-7-phosphoheptulonate synthase
aroB			LBUCD034_RS11850		CG419_RS05420		lp_1086	L0060	LEUM_RS05690	K01735	3-dehydroquinate synthase
aroKB										K13829	Shikimate kinase / 3-dehydroquinate synthase
aroD			LBUCD034_RS01315		CG419_RS05380		lp_3493	L0062	LEUM_RS05660	K03785	3-dehydroquinate dehydratase I
aroE			LBUCD034_RS01360	LBCZ_RS01660	CG419_RS05410		lp_3494	L0061	LEUM_RS05700	K00014	Shikimate dehydrogenase
aroE				LBCZ_RS01670			lp_3498			K00014	Shikimate dehydrogenase
aroE							lp_3499			K00014	Shikimate dehydrogenase
aroK|aroL			LBUCD034_RS01355		CG419_RS05425	LAF_RS06345	lp_2033	L0056	LEUM_RS05670	K00891	Shikimate kinase
aroA			LBUCD034_RS01345			LAF_RS06355	lp_2035	L0057	LEUM_RS05680	K00800	3-phosphoshikimate 1-carboxyvinyltransferase
aroC			LBUCD034_RS01340			LAF_RS06365	lp_2037	L0059	LEUM_RS05685	K01736	Chorismate synthase
trpE			LBUCD034_RS11860				lp_1652	L0054	LEUM_RS05715	K01657	Anthranilate synthase component I
trpG			LBUCD034_RS11865					L0178	LEUM_RS05710	K01658	Anthranilate synthase component II
trpD			LBUCD034_RS11195				lp_1654	L0052	LEUM_RS05735	K00766	Anthranilate phosphoribosyltransferase
trpF			LBUCD034_RS11205				lp_1656	L0050	LEUM_RS05725	K01817	Phosphoribosylanthranilate isomerase
trpF									LEUM_RS09800	K01817	Phosphoribosylanthranilate isomerase
trpC			LBUCD034_RS11200				lp_1655	L0051	LEUM_RS05730	K01609	Indole-3-glycerol phosphate synthase
trpA			LBUCD034_RS11215				lp_1658	L0048	LEUM_RS05720	K01695	Tryptophan synthase alpha chain
trpB			LBUCD034_RS11210				lp_1657	L0049	LEUM_RS05740	K01696	Tryptophan synthase beta chain
**Phenylalanine**											
aroF|aroG|aroH								L0063		K01626	3-deoxy-7-phosphoheptulonate synthase
aroF|aroG|aroH								L0064		K01626	3-deoxy-7-phosphoheptulonate synthase
AROA2|aroA			LBUCD034_RS11855		CG419_RS05415		lp_1085		LEUM_RS05695	K03856	3-deoxy-7-phosphoheptulonate synthase
aroB			LBUCD034_RS11850		CG419_RS05420		lp_1086	L0060	LEUM_RS05690	K01735	3-dehydroquinate synthase
aroD			LBUCD034_RS01315		CG419_RS05380		lp_3493	L0062	LEUM_RS05660	K03785	3-dehydroquinate dehydratase I
aroE			LBUCD034_RS01360	LBCZ_RS01660	CG419_RS05410		lp_3494	L0061	LEUM_RS05700	K00014	Shikimate dehydrogenase
aroE				LBCZ_RS01670			lp_3498			K00014	Shikimate dehydrogenase
aroE							lp_3499			K00014	Shikimate dehydrogenase
aroK|aroL			LBUCD034_RS01355		CG419_RS05425	LAF_RS06345	lp_2033	L0056	LEUM_RS05670	K00891	Shikimate kinase
aroA			LBUCD034_RS01345			LAF_RS06355	lp_2035	L0057	LEUM_RS05680	K00800	3-phosphoshikimate 1-carboxyvinyltransferase
aroC			LBUCD034_RS01340			LAF_RS06365	lp_2037	L0059	LEUM_RS05685	K01736	Chorismate synthase
pheA						LAF_RS06370				K14170	Chorismate mutase / prephenate dehydratase
AROA1|aroA			LBUCD034_RS01335		CG419_RS01860			L64445		K04516	Chorismate mutase
pheB			LBUCD034_RS11680							K06209	Chorismate mutase
pheA2			LBUCD034_RS01065					L0055		K04518	Prephenate dehydratase
pheA						LAF_RS06370				K14170	Chorismate mutase / prephenate dehydratase
aspB		LVIS_RS15700	LBUCD034_RS05885	LBCZ_RS06920		LAF_RS06085	lp_1739	L0098		K00812	Aspartate aminotransferase
aspB										K00812	Aspartate aminotransferase
hisC			LBUCD034_RS03690	LBCZ_RS06660	CG419_RS06545	LAF_RS04495	lp_2551	L0065	LEUM_RS07570	K00817	Histidinol-phosphate aminotransferase
tyrB				LBCZ_RS10030						K00832	Aromatic-amino-acid transaminase
**Tyrosine**											
aroF|aroG|aroH								L0063		K01626	3-deoxy-7-phosphoheptulonate synthase
aroF|aroG|aroH								L0064		K01626	3-deoxy-7-phosphoheptulonate synthase
AROA2|aroA			LBUCD034_RS11855		CG419_RS05415		lp_1085		LEUM_RS05695	K03856	3-deoxy-7-phosphoheptulonate synthase
aroB			LBUCD034_RS11850		CG419_RS05420		lp_1086	L0060	LEUM_RS05690	K01735	3-dehydroquinate synthase
aroD			LBUCD034_RS01315		CG419_RS05380		lp_3493	L0062	LEUM_RS05660	K03785	3-dehydroquinate dehydratase I
aroE			LBUCD034_RS01360	LBCZ_RS01660	CG419_RS05410		lp_3494	L0061	LEUM_RS05700	K00014	Shikimate dehydrogenase
aroE				LBCZ_RS01670			lp_3498			K00014	Shikimate dehydrogenase
aroE							lp_3499			K00014	Shikimate dehydrogenase
aroK|aroL			LBUCD034_RS01355		CG419_RS05425	LAF_RS06345	lp_2033	L0056	LEUM_RS05670	K00891	Shikimate kinase
aroKB										K13829	Shikimate kinase / 3-dehydroquinate synthase
aroA			LBUCD034_RS01345			LAF_RS06355	lp_2035	L0057	LEUM_RS05680	K00800	3-phosphoshikimate 1-carboxyvinyltransferase
aroC			LBUCD034_RS01340			LAF_RS06365	lp_2037	L0059	LEUM_RS05685	K01736	Chorismate synthase
pheA						LAF_RS06370				K14170	Chorismate mutase / prephenate dehydratase
AROA1|aroA			LBUCD034_RS01335		CG419_RS01860			L64445		K04516	Chorismate mutase
pheB			LBUCD034_RS11680							K06209	Chorismate mutase
tyrA2			LBUCD034_RS01350			LAF_RS06350	lp_2034	L0058	LEUM_RS05675	K04517	Prephenate dehydrogenase
aspB		LVIS_RS15700	LBUCD034_RS05885	LBCZ_RS06920		LAF_RS06085	lp_1739	L0098		K00812	Aspartate aminotransferase
aspB										K00812	Aspartate aminotransferase
hisC			LBUCD034_RS03690	LBCZ_RS06660	CG419_RS06545	LAF_RS04495	lp_2551	L0065	LEUM_RS07570	K00817	Histidinol-phosphate aminotransferase
tyrB				LBCZ_RS10030						K00832	Aromatic-amino-acid transaminase
**Isoleucine**											
ilvB|ilvG|ilvI		LVIS_RS13945	LBUCD034_RS00260	LBCZ_RS08845	CG419_RS05895	LAF_RS05895	lp_3587	L210	LEUM_RS02545	K01652	Acetolactate synthase I/II/III large subunit
ilvB|ilvG|ilvI			LBUCD034_RS11775					L0078	LEUM_RS09540	K01652	Acetolactate synthase I/II/III large subunit

Gene	Other name(s)	*Lb. brevis* ATCC 367	*Lb. buchneri* CD034	*Lb. casei* ATCC 393	*Lb. curvatus* MRS6	*Lb. fermentum* IFO 3956	*Lb. plantarum* WCFS1	*L. lactis* IL1403	*Leu. mesenteroides* ATCC 8293	KEGG ID	Enzyme full name
ilvH|ilvN								L0079	LEUM_RS09535	K01653	Acetolactate synthase I/III small subunit
ilvC								L0080	LEUM_RS09530	K00053	Ketol-acid reductoisomerase
ilvC										K00053	Ketol-acid reductoisomerase
ilvD								L0077	LEUM_RS09930	K01687	Dihydroxy-acid dehydratase
ilvE			LBUCD034_RS03635	LBCZ_RS09375		LAF_RS02015	lp_2390	L0086	LEUM_RS06440	K00826	Branched-chain amino acid aminotransferase
ilvE			LBUCD034_RS03640			LAF_RS05355				K00826	Branched-chain amino acid aminotransferase
**Valine**											
ilvB|ilvG|ilvI		LVIS_RS13945	LBUCD034_RS00260	LBCZ_RS08845	CG419_RS05895	LAF_RS05895	lp_3587	L210	LEUM_RS02545	K01652	Acetolactate synthase I/II/III large subunit
ilvB|ilvG|ilvI			LBUCD034_RS11775					L0078	LEUM_RS09540	K01652	Acetolactate synthase I/II/III large subunit
ilvH|ilvN								L0079	LEUM_RS09535	K01653	Acetolactate synthase I/III small subunit
ilvC								L0080	LEUM_RS09530	K00053	Ketol-acid reductoisomerase
ilvC										K00053	Ketol-acid reductoisomerase
ilvD								L0077	LEUM_RS09930	K01687	Dihydroxy-acid dehydratase
ilvE			LBUCD034_RS03635	LBCZ_RS09375		LAF_RS02015	lp_2390	L0086	LEUM_RS06440	K00826	Branched-chain amino acid aminotransferase
ilvE			LBUCD034_RS03640			LAF_RS05355				K00826	Branched-chain amino acid aminotransferase
**Leucine**											
ilvB|ilvG|ilvI		LVIS_RS13945	LBUCD034_RS00260	LBCZ_RS08845	CG419_RS05895	LAF_RS05895	lp_3587	L210	LEUM_RS02545	K01652	Acetolactate synthase I/II/III large subunit
ilvB|ilvG|ilvI			LBUCD034_RS11775					L0078	LEUM_RS09540	K01652	Acetolactate synthase I/II/III large subunit
ilvH|ilvN								L0079	LEUM_RS09535	K01653	Acetolactate synthase I/III small subunit
ilvC								L0080	LEUM_RS09530	K00053	Ketol-acid reductoisomerase
ilvC										K00053	Ketol-acid reductoisomerase
ilvD								L0077	LEUM_RS09930	K01687	Dihydroxy-acid dehydratase
leuA			LBUCD034_RS00335		CG419_RS09190		lp_3476		LEUM_RS09890	K01649	2-isopropylmalate synthase
leuC						LAF_RS05585		L0075	LEUM_RS09880	K01703	3-isopropylmalate/(R)-2-methylmalate dehydratase large subunit
leuD						LAF_RS05580		L0076	LEUM_RS09875	K01704	3-isopropylmalate/(R)-2-methylmalate dehydratase small subunit
leuB						LAF_RS05590		L0074	LEUM_RS09885	K00052	3-isopropylmalate dehydrogenase
ilvE			LBUCD034_RS03635	LBCZ_RS09375		LAF_RS02015	lp_2390	L0086	LEUM_RS06440	K00826	Branched-chain amino acid aminotransferase
ilvE			LBUCD034_RS03640			LAF_RS05355				K00826	Branched-chain amino acid aminotransferase
**Glutamate**											
gltB								L0119	LEUM_RS03985	K00265	Glutamate synthase (NADPH) large chain
gltD								L114827	LEUM_RS03990	K00266	Glutamate synthase (NADPH) small chain
glnA|GLUL		LVIS_RS16370	LBUCD034_RS06320	LBCZ_RS07845	CG419_RS03570	LAF_RS07240	lp_1581	L0118	LEUM_RS03495	K01915	Glutamine synthetase
glnA|GLUL				LBCZ_RS02945						K01915	Glutamine synthetase
**Proline**											
argJ			LBUCD034_RS09000					L0105	LEUM_RS00300	K00620	Glutamate N-acetyltransferase / amino-acid N-acetyltransferase
argB			LBUCD034_RS08995			LAF_RS04255	lp_0530	L0107	LEUM_RS00295	K00930	Acetylglutamate kinase
argC			LBUCD034_RS09005			LAF_RS04265	lp_0487	L0104	LEUM_RS00305	K00145	N-acetyl-gamma-glutamyl-phosphate reductase
argD			LBUCD034_RS08990			LAF_RS04250	lp_0531	L0106	LEUM_RS00290	K00818	Acetylornithine aminotransferase
argE			LBUCD034_RS00425					L0115		K01438	Acetylornithine deacetylase
argE			LBUCD034_RS11875							K01438	Acetylornithine deacetylase
ocd						LAF_RS05385				K01750	Ornithine cyclodeaminase
proB			LBUCD034_RS10125	LBCZ_RS10930		LAF_RS07660	lp_0016	L0117	LEUM_RS01420	K00931	Glutamate 5-kinase
proA			LBUCD034_RS10130	LBCZ_RS10925		LAF_RS07655	lp_0017	L0116	LEUM_RS01425	K00147	Glutamate-5-semialdehyde dehydrogenase
proC		LVIS_RS14165	LBUCD034_RS07595	LBCZ_RS08665	CG419_RS02240	LAF_RS07910	lp_0561	L135991	LEUM_RS09075	K00286	Pyrroline-5-carboxylate reductase
**Arginine**											
argJ			LBUCD034_RS09000					L0105	LEUM_RS00300	K00620	Glutamate N-acetyltransferase / amino-acid N-acetyltransferase
argB			LBUCD034_RS08995			LAF_RS04255	lp_0530	L0107	LEUM_RS00295	K00930	Acetylglutamate kinase
argC			LBUCD034_RS09005			LAF_RS04265	lp_0487	L0104	LEUM_RS00305	K00145	N-acetyl-gamma-glutamyl-phosphate reductase
argD			LBUCD034_RS08990			LAF_RS04250	lp_0531	L0106	LEUM_RS00290	K00818	Acetylornithine aminotransferase
argE			LBUCD034_RS00425					L0115		K01438	Acetylornithine deacetylase
argE			LBUCD034_RS11875							K01438	Acetylornithine deacetylase
OTC|argF|argI		LVIS_RS21430	LBUCD034_RS02395			LAF_RS01760	lp_0532	L0108	LEUM_RS07120	K00611	Ornithine carbamoyltransferase
OTC|argF|argI								L0109		K00611	Ornithine carbamoyltransferase
argG|ASS1			LBUCD034_RS03905	LBCZ_RS13220		LAF_RS09265	lp_0775	L126739	LEUM_RS07130	K01940	Argininosuccinate synthase
argH|ASL			LBUCD034_RS03910	LBCZ_RS13225		LAF_RS09260	lp_0776	L0114	LEUM_RS07125	K01755	Argininosuccinate lyase
rocF|arg						LAF_RS09330				K01476	Arginase
arcA		LVIS_RS21435	LBUCD034_RS02390			LAF_RS01770		L0329		K01478	Arginine deiminase
**Putrescine**											
argJ			LBUCD034_RS09000					L0105	LEUM_RS00300	K00620	Glutamate N-acetyltransferase / amino-acid N-acetyltransferase
argB			LBUCD034_RS08995			LAF_RS04255	lp_0530	L0107	LEUM_RS00295	K00930	Acetylglutamate kinase
argC			LBUCD034_RS09005			LAF_RS04265	lp_0487	L0104	LEUM_RS00305	K00145	N-acetyl-gamma-glutamyl-phosphate reductase
argD			LBUCD034_RS08990			LAF_RS04250	lp_0531	L0106	LEUM_RS00290	K00818	Acetylornithine aminotransferase
argE			LBUCD034_RS00425					L0115		K01438	Acetylornithine deacetylase
argE			LBUCD034_RS11875							K01438	Acetylornithine deacetylase
ODC1|speC|speF				LBCZ_RS07855						K01581	Ornithine decarboxylase
**4-Aminobutanoate (GABA)**											
gadB|gadA|GAD		LVIS_RS11930					lp_3420	L123581		K01580	Glutamate decarboxylase
gadB|gadA|GAD		LVIS_RS20565								K01580	Glutamate decarboxylase
**Inosine monophosphate (IMP)**											
tktA|tktB		LVIS_RS18460				LAF_RS09780	lp_0489	L0043	LEUM_RS05705	K00615	Transketolase
tktA|tktB							lp_1083			K00615	Transketolase

Gene	Other name(s)	*Lb. brevis* ATCC 367	*Lb. buchneri* CD034	*Lb. casei* ATCC 393	*Lb. curvatus* MRS6	*Lb. fermentum* IFO 3956	*Lb. plantarum* WCFS1	*L. lactis* IL1403	*Leu. mesenteroides* ATCC 8293	KEGG ID	Enzyme full name
tktA|tktB							lp_3135			K00615	Transketolase
tktA|tktB							lp_3538			K00615	Transketolase
PRPS|prsA		LVIS_RS13875	LBUCD034_RS08065	LBCZ_RS12200	CG419_RS01975	LAF_RS01120	lp_0471	L25614	LEUM_RS03400	K00948	Ribose-phosphate pyrophosphokinase
PRPS|prsA							lp_2166			K00948	Ribose-phosphate pyrophosphokinase
purF|PPAT			LBUCD034_RS01940	LBCZ_RS08420	CG419_RS07140	LAF_RS00690	lp_2723	L171350	LEUM_RS03530	K00764	Amidophosphoribosyltransferase
purD			LBUCD034_RS01960	LBCZ_RS08400	CG419_RS07120	LAF_RS00710	lp_2719	L153005	LEUM_RS03415	K01945	Phosphoribosylamine---glycine ligase
purN			LBUCD034_RS01950	LBCZ_RS08410	CG419_RS07130	LAF_RS00700	lp_2721	L164626	LEUM_RS03540	K11175	Phosphoribosylglycinamide formyltransferase 1
purT										K08289	Phosphoribosylglycinamide formyltransferase 2
purL|PFAS								L177031		K01952	Phosphoribosylformylglycinamidine synthase
purL|PFAS			LBUCD034_RS01925	LBCZ_RS08435	CG419_RS07155	LAF_RS00675	lp_2726		LEUM_RS03515	K01952	Phosphoribosylformylglycinamidine synthase
purL|PFAS			LBUCD034_RS01930	LBCZ_RS08430	CG419_RS07150	LAF_RS00680			LEUM_RS03520	K01952	Phosphoribosylformylglycinamidine synthase
purL|PFAS			LBUCD034_RS01935	LBCZ_RS08425	CG419_RS07145	LAF_RS00685			LEUM_RS03525	K01952	Phosphoribosylformylglycinamidine synthase
purL|PFAS							lp_2725	L176360		K01952	Phosphoribosylformylglycinamidine synthase
purL|PFAS							lp_2724	L173921		K01952	Phosphoribosylformylglycinamidine synthase
purM			LBUCD034_RS01945	LBCZ_RS08415	CG419_RS07135	LAF_RS00695	lp_2722	L165202		K01933	Phosphoribosylformylglycinamidine cyclo-ligase
purM		LVIS_RS21965	LBUCD034_RS11510							K01933	Phosphoribosylformylglycinamidine cyclo-ligase
purM			LBUCD034_RS11590							K01933	Phosphoribosylformylglycinamidine cyclo-ligase
purM									LEUM_RS03535	K01933	Phosphoribosylformylglycinamidine cyclo-ligase
purM									LEUM_RS09635	K01933	Phosphoribosylformylglycinamidine cyclo-ligase
purK			LBUCD034_RS01915	LBCZ_RS08445		LAF_RS00645	lp_2728	L151330	LEUM_RS03505	K01589	5-(carboxyamino)imidazole ribonucleotide synthase
purK		LVIS_RS19515	LBUCD034_RS08570	LBCZ_RS05220	CG419_RS02420	LAF_RS08420				K01589	5-(carboxyamino)imidazole ribonucleotide synthase
purK							lp_1141			K01589	5-(carboxyamino)imidazole ribonucleotide synthase
purE			LBUCD034_RS01910	LBCZ_RS08450	CG419_RS07170	LAF_RS00640	lp_2729	L152487	LEUM_RS03500	K01588	5-(carboxyamino)imidazole ribonucleotide mutase
purC			LBUCD034_RS01920	LBCZ_RS08440	CG419_RS07160	LAF_RS00670	lp_2727	L177350	LEUM_RS03510	K01923	Phosphoribosylaminoimidazole-succinocarboxamide synthase
purB|AD[illegible]		LVIS_RS12650	LBUCD034_RS02605	LBCZ_RS05225	CG419_RS02425	LAF_RS00650	lp_3269	L88187	LEUM_RS06785	K01756	Adenylosuccinate lyase
purH			LBUCD034_RS01955	LBCZ_RS08405	CG419_RS07125	LAF_RS00705	lp_2720	L158710	LEUM_RS03545	K00602	Phosphoribosylaminoimidazolecarboxamide formyltransferase / IMP cyclohydrolase
**Guanosine monophosphate (GMP)**											
tktA|tktB		LVIS_RS18460				LAF_RS09780	lp_0489	L0043	LEUM_RS05705	K00615	Transketolase
tktA|tktB							lp_1083			K00615	Transketolase
tktA|tktB							lp_3135			K00615	Transketolase
tktA|tktB							lp_3538			K00615	Transketolase
PRPS|prsA		LVIS_RS13875	LBUCD034_RS08065	LBCZ_RS12200	CG419_RS01975	LAF_RS01120	lp_0471	L25614	LEUM_RS03400	K00948	Ribose-phosphate pyrophosphokinase
PRPS|prsA							lp_2166			K00948	Ribose-phosphate pyrophosphokinase
purF|PPAT			LBUCD034_RS01940	LBCZ_RS08420	CG419_RS07140	LAF_RS00690	lp_2723	L171350	LEUM_RS03530	K00764	Amidophosphoribosyltransferase
purD			LBUCD034_RS01960	LBCZ_RS08400	CG419_RS07120	LAF_RS00710	lp_2719	L153005	LEUM_RS03415	K01945	Phosphoribosylamine---glycine ligase
purN			LBUCD034_RS01950	LBCZ_RS08410	CG419_RS07130	LAF_RS00700	lp_2721	L164626	LEUM_RS03540	K11175	Phosphoribosylglycinamide formyltransferase 1
purT										K08289	Phosphoribosylglycinamide formyltransferase 2
purL|PFAS								L177031		K01952	Phosphoribosylformylglycinamidine synthase
purL|PFAS			LBUCD034_RS01925	LBCZ_RS08435	CG419_RS07155	LAF_RS00675	lp_2726		LEUM_RS03515	K01952	Phosphoribosylformylglycinamidine synthase
purL|PFAS			LBUCD034_RS01930	LBCZ_RS08430	CG419_RS07150	LAF_RS00680			LEUM_RS03520	K01952	Phosphoribosylformylglycinamidine synthase
purL|PFAS			LBUCD034_RS01935	LBCZ_RS08425	CG419_RS07145	LAF_RS00685			LEUM_RS03525	K01952	Phosphoribosylformylglycinamidine synthase
purL|PFAS							lp_2725	L176360		K01952	Phosphoribosylformylglycinamidine synthase
purL|PFAS							lp_2724	L173921		K01952	Phosphoribosylformylglycinamidine synthase
purM			LBUCD034_RS01945	LBCZ_RS08415	CG419_RS07135	LAF_RS00695	lp_2722	L165202		K01933	Phosphoribosylformylglycinamidine cyclo-ligase
purM		LVIS_RS21965	LBUCD034_RS11510							K01933	Phosphoribosylformylglycinamidine cyclo-ligase
purM			LBUCD034_RS11590							K01933	Phosphoribosylformylglycinamidine cyclo-ligase
purM									LEUM_RS03535	K01933	Phosphoribosylformylglycinamidine cyclo-ligase
purM									LEUM_RS09635	K01933	Phosphoribosylformylglycinamidine cyclo-ligase
purK			LBUCD034_RS01915	LBCZ_RS08445		LAF_RS00645	lp_2728	L151330	LEUM_RS03505	K01589	5-(carboxyamino)imidazole ribonucleotide synthase
purK		LVIS_RS19515	LBUCD034_RS08570	LBCZ_RS05220	CG419_RS02420	LAF_RS08420				K01589	5-(carboxyamino)imidazole ribonucleotide synthase
purK							lp_1141			K01589	5-(carboxyamino)imidazole ribonucleotide synthase
purE			LBUCD034_RS01910	LBCZ_RS08450	CG419_RS07170	LAF_RS00640	lp_2729	L152487	LEUM_RS03500	K01588	5-(carboxyamino)imidazole ribonucleotide mutase
purC			LBUCD034_RS01920	LBCZ_RS08440	CG419_RS07160	LAF_RS00670	lp_2727	L177350	LEUM_RS03510	K01923	Phosphoribosylaminoimidazole-succinocarboxamide synthase
purB|ADSL		LVIS_RS12650	LBUCD034_RS02605	LBCZ_RS05225	CG419_RS02425	LAF_RS00650	lp_3269	L88187	LEUM_RS06785	K01756	Adenylosuccinate lyase
purH			LBUCD034_RS01955	LBCZ_RS08405	CG419_RS07125	LAF_RS00705	lp_2720	L158710	LEUM_RS03545	K00602	Phosphoribosylaminoimidazolecarboxamide formyltransferase / IMP cyclohydrolase
guaB				LBCZ_RS00770	CG419_RS08905		lp_3194	L21264	LEUM_RS03155	K00088	IMP dehydrogenase
guaB									LEUM_RS03160	K00088	IMP dehydrogenase
guaB		LVIS_RS21590	LBUCD034_RS02930			LAF_RS00435				K00088	IMP dehydrogenase
guaA		LVIS_RS20130	LBUCD034_RS09200	LBCZ_RS09055	CG419_RS09980		lp_0914	L115968		K01951	GMP synthase (glutamine-hydrolysing)
guaA										K01951	GMP synthase (glutamine-hydrolysing)
guaA		LVIS_RS22460								K01951	GMP synthase (glutamine-hydrolysing)
guaA			LBUCD034_RS09335	LBCZ_RS10310		LAF_RS04890	lp_0073			K01951	GMP synthase (glutamine-hydrolysing)
guaA									LEUM_RS03165	K01951	GMP synthase (glutamine-hydrolysing)
**Adenosine monophosphate (AMP)**											
tktA|tktB		LVIS_RS18460				LAF_RS09780	lp_0489	L0043	LEUM_RS05705	K00615	Transketolase
tktA|tktB							lp_1083			K00615	Transketolase
tktA|tktB							lp_3135			K00615	Transketolase
tktA|tktB							lp_3538			K00615	Transketolase
PRPS|prsA		LVIS_RS13875	LBUCD034_RS08065	LBCZ_RS12200	CG419_RS01975	LAF_RS01120	lp_0471	L25614	LEUM_RS03400	K00948	Ribose-phosphate pyrophosphokinase
PRPS|prsA							lp_2166			K00948	Ribose-phosphate pyrophosphokinase
purF|PPAT			LBUCD034_RS01940	LBCZ_RS08420	CG419_RS07140	LAF_RS00690	lp_2723	L171350	LEUM_RS03530	K00764	Amidophosphoribosyltransferase

Gene	Other name(s)	*Lb. brevis* ATCC 367	*Lb. buchneri* CD034	*Lb. casei* ATCC 393	*Lb. curvatus* MRS6	*Lb. fermentum* IFO 3956	*Lb. plantarum* WCFS1	*L. lactis* IL1403	*Leu. mesenteroides* ATCC 8293	KEGG ID	Enzyme full name
purD			LBUCD034_RS01960	LBCZ_RS08400	CG419_RS07120	LAF_RS00710	lp_2719	L153005	LEUM_RS03415	K01945	Phosphoribosylamine---glycine ligase
purN			LBUCD034_RS01950	LBCZ_RS08410	CG419_RS07130	LAF_RS00700	lp_2721	L164626	LEUM_RS03540	K11175	Phosphoribosylglycinamide formyltransferase 1
purT										K08289	Phosphoribosylglycinamide formyltransferase 2
purL|PFAS								L177031		K01952	Phosphoribosylformylglycinamidine synthase
purL|PFAS			LBUCD034_RS01925	LBCZ_RS08435	CG419_RS07155	LAF_RS00675	lp_2726		LEUM_RS03515	K01952	Phosphoribosylformylglycinamidine synthase
purL|PFAS			LBUCD034_RS01930	LBCZ_RS08430	CG419_RS07150	LAF_RS00680			LEUM_RS03520	K01952	Phosphoribosylformylglycinamidine synthase
purL|PFAS			LBUCD034_RS01935	LBCZ_RS08425	CG419_RS07145	LAF_RS00685			LEUM_RS03525	K01952	Phosphoribosylformylglycinamidine synthase
purL|PFAS							lp_2725	L176360		K01952	Phosphoribosylformylglycinamidine synthase
purL|PFAS							lp_2724	L173921		K01952	Phosphoribosylformylglycinamidine synthase
purM			LBUCD034_RS01945	LBCZ_RS08415	CG419_RS07135	LAF_RS00695	lp_2722	L165202		K01933	Phosphoribosylformylglycinamidine cyclo-ligase
purM		LVIS_RS21965	LBUCD034_RS11510							K01933	Phosphoribosylformylglycinamidine cyclo-ligase
purM			LBUCD034_RS11590							K01933	Phosphoribosylformylglycinamidine cyclo-ligase
purM									LEUM_RS03535	K01933	Phosphoribosylformylglycinamidine cyclo-ligase
purM									LEUM_RS09635	K01933	Phosphoribosylformylglycinamidine cyclo-ligase
purK			LBUCD034_RS01915	LBCZ_RS08445		LAF_RS00645	lp_2728	L151330	LEUM_RS03505	K01589	5-(carboxyamino)imidazole ribonucleotide synthase
purK		LVIS_RS19515	LBUCD034_RS08570	LBCZ_RS05220	CG419_RS02420	LAF_RS08420				K01589	5-(carboxyamino)imidazole ribonucleotide synthase
purK							lp_1141			K01589	5-(carboxyamino)imidazole ribonucleotide synthase
purE			LBUCD034_RS01910	LBCZ_RS08450	CG419_RS07170	LAF_RS00640	lp_2729	L152487	LEUM_RS03500	K01588	5-(carboxyamino)imidazole ribonucleotide mutase
purC			LBUCD034_RS01920	LBCZ_RS08440	CG419_RS07160	LAF_RS00670	lp_2727	L177350	LEUM_RS03510	K01923	Phosphoribosylaminoimidazole-succinocarboxamide synthase
purH			LBUCD034_RS01955	LBCZ_RS08405	CG419_RS07125	LAF_RS00705	lp_2720	L158710	LEUM_RS03545	K00602	Phosphoribosylaminoimidazolecarboxamide formyltransferase / IMP cyclohydrolase
purA		LVIS_RS12645	LBUCD034_RS02600	LBCZ_RS00310	CG419_RS00340	LAF_RS00225	lp_3270	L12179		K01939	Adenylosuccinate synthase
purA									LEUM_RS07135	K01939	Adenylosuccinate synthase
purB		LVIS_RS12650	LBUCD034_RS02605	LBCZ_RS05225	CG419_RS02425	LAF_RS00650	lp_3269	L88187	LEUM_RS06785	K01756	Adenylosuccinate lyase
purB			LBUCD034_RS02175							K01756	Adenylosuccinate lyase
